# Predicting Urban Reservoir Levels Using Statistical Learning Techniques

**DOI:** 10.1038/s41598-018-23509-w

**Published:** 2018-03-26

**Authors:** Renee Obringer, Roshanak Nateghi

**Affiliations:** 1 Department of Earth, Atmospheric, and Planetary Sciences, Purdue University, West Lafayette, IN 47907 USA; 2 School of Industrial Engineering, and Division of Environmental and Ecological Engineering, Purdue University, West Lafayette, IN 47907 USA

## Abstract

Urban water supplies are critical to the growth of the city and the wellbeing of its citizens. However, these supplies can be vulnerable to hydrological extremes, such as droughts and floods, especially if they are the main source of water for the city. Maintaining these supplies and preparing for future conditions is a crucial task for water managers, but predicting hydrological extremes is a challenge. This study tested the abilities of eight statistical learning techniques to predict reservoir levels, given the current hydroclimatic conditions, and provide inferences on the key predictors of reservoir levels. The results showed that random forest, an ensemble, tree-based method, was the best algorithm for predicting reservoir levels. We initially developed the models using Lake Sidney Lanier (Atlanta, Georgia) as the test site; however, further analysis demonstrated that the model based on the random forest algorithm was transferable to other reservoirs, specifically Eagle Creek (Indianapolis, Indiana) and Lake Travis (Austin, Texas). Additionally, we found that although each reservoir was impacted differently, streamflow, city population, and El Niño/Southern Oscillation (ENSO) index were repeatedly among the most important predictors. These are critical variables which can be used by water managers to recognize the potential for reservoir level changes.

## Introduction

One of the major tasks for urban water managers is maintaining the reservoirs that provide the city’s drinking water as well as reacting to changes brought on by various hydroclimatic conditions. For example, after a large rain event, water may need to be released downstream to avoid flooding, and during a long-term drought, water use restrictions may need to be implemented to reduce the impact of the drought and conserve water. Preparation for these events is key if one wants to reduce the impacts of flooding or water stress, both of which can cause major ecological, economic, and societal problems. Understanding and predicting urban floods and droughts, often referred to as hydrological droughts^1^, is a major focus of the urban resilience community. An important step to improve urban resilience is to understand and predict urban reservoir responses under the various hydroclimatic conditions that lead to flooding and droughts, so that water managers can implement the necessary mitigation policies (e.g., controlled releases or water use restrictions). Moreover, urban water supplies are especially at-risk to future hydrological extremes because of the unprecedented urban growth that is happening around the world. Currently, about 50% of the world population lives in cities, and the World Bank has projected that by 2050, this number will grow to 65%^2^. When paired with a changing hydrological environment, including an increased likelihood of droughts^3^, rapid urban growth puts cities and their watersheds in a vulnerable position.

To minimize these vulnerabilities, water managers must be aware of the likelihood of any major changes in reservoir level that may affect water availability, so that they can begin to prepare and, hopefully, minimize any negative effects. The typical approach to predicting hydrological extremes in reservoirs is through probabilistic analyses. Most notably, de Araújo and Bronstert used a simple volume equation to assess changes in a Brazilian reservoir^4^. They included inputs such as precipitation and streamflow and outputs such as withdrawals and infiltration. This analysis demonstrated the correlation between reservoir level and drought severity. That is, as the drought increases in severity, the reservoir levels decrease. De Araújo and Bronstert also found that small, isolated systems cannot cope with long-term droughts, making it important for cities with these systems to be proactive in their drought planning. Finally, the authors found that hydrological droughts are often out of phase with meteorological droughts, which provides evidence towards the need to evaluate droughts in reservoirs separately than the typical meteorological (precipitation-based) droughts if we are to improve urban water system resilience.

There are a few studies that have gone beyond the basic volume equation and done predictive studies on reservoirs, including that of Ficchi *et al*. This study focused on predicting reservoir levels for flood applications on the Seine River^5^, where there are several small reservoirs that are designed to control the streamflow and prevent flooding downstream. Prior to this study, the reservoirs were managed based on historical averages, however, when the streamflow was significantly different than the average, this method failed to prevent flooding downstream. The authors leveraged a tree-based model that used weather data from the European Centre for Medium-Range Weather Forecasts (ECMWF) as the input to predict water levels. They found that the model could adequately predict high-flow conditions within the next 9 days, which would allow water managers to implement the regulating features and therefore reduce the risk of flooding downstream. However, when they repeated their analysis for low-flow scenarios, the authors found that they could not accurately predict droughts, likely because droughts require longer forecasts, which cannot always be made with the meteorological data used in this study. Similar work was done by Yang *et al*. on reservoir discharges in California^6^. In this study, the authors used two different types of tree-based algorithms: classification and regression trees (CART) and random forest, to predict the outflow of the reservoirs. The outflow in this study is considered the controlled release of water back into the river if the reservoir levels get too high. The results showed that random forest was able to successfully predict when controlled releases should occur, based on the reservoir storage, precipitation, reservoir inflows, runoff, snowpack, and downstream river conditions. Additionally, the authors leveraged cross-validation to avoid overfitting of the model. The predictions of the cross-validated model outperformed the basic run, demonstrating the importance of performing cross-validation during the model selection process. Finally, the authors showed that random forest was also able to predict the storage trajectory of the reservoirs, an encouraging result for our present study.

Although these predictive studies demonstrated the ability of supervised learning techniques to accurately predict reservoir conditions, the authors were mostly interested in the short-term high-flow conditions that are indicative of floods. The work presented here focuses on all hydroclimatic conditions, including those related to floods and those to droughts. Additionally, the previous work on the subject mainly focused on tree-based models. In this study, using readily available hydroclimatic data, we built several predictive models to determine the reservoir levels based on the current conditions. The knowledge of future reservoir levels, even if it is not exact, is crucial for water managers and policymakers, who must decide when to implement various flood- and drought-related regulations.

The objectives of this study were to test the performance of different statistical learning techniques in predicting the water levels in Lake Lanier (Atlanta, Georgia, USA) based on the current hydroclimatic conditions and city characteristics, and to determine the best model for the task. We hypothesized that the random forest model would perform the best, as both Ficchi *et al*. and Yang *et al*. demonstrated the ability of tree-based models to predict reservoir levels^5,6^.

## Results

### Predictive performance of the statistical models

Supervised learning theory is a branch of statistical learning methods that has been extensively applied to areas ranging from risk and resilience analysis to hydrological modeling^7–10^. Supervised learning models vary widely in their degree of complexity, stability, flexibility and interpretability, and can be categorized as parametric, semi-parametric or non-parametric methods. The most popular approach is parametric modeling (e.g., generalized linear regression models) where a parametric function is fitted to the training data (e.g., via mechanisms such as least-squares), such that: $\hat{f}({\boldsymbol{X}})=g({\boldsymbol{X}}|{\{{\hat{\beta }}_{j}\}}_{1}^{p})$. The advantage of parametric modeling is that by assuming a functional form, estimating the complex shape of the response function can be simplified as estimating a set of *β* parameters, which renders the method simple to compute and interpret. However, such an approach is ‘inflexible’ and often fails to approximate the true function accurately (since the dependencies in real data are rarely linear). *Non-parametric* models, on the other hand, do not make assumptions about the shape of the function *f*. Instead, they harness the power of the input data to approximate the function. While they have the advantage of *not* assuming unrealistic functional form and thereby better approximating the true function, they can be very data-intensive^11^.

In this study, we assessed the predictive performance of eight different statistical learning models—generalized linear model (GLM)^12^, generalized additive model (GAM)^13^, multivariate adaptive regression splines (MARS)^11^, classification and regression tree (CART)^14^, bagged CART^15^, random forest^16^, support vector machine (SVM)^17^, and Bayesian additive regression tree (BART)^18^—to predict the reservoir level based on the hydroclimatic conditions at that time. These eight models were chosen in order to obtain a wide variety of parametric, semi-parametric and non-parametric algorithms to test. We included linear models such as GLM and more complex additive models such as GAM and MARS, tree-based models, such as CART, random forest and BART, and more complex data-miners, such as SVM. In this way, we can ensure that we have tested the performance of a wide range of statistical learning algorithms, and not limited to the scope of tree-based models alone. The rational for including a linear parametric model such as generalized linear models is that, as described above, they are highly interpretable and lend themselves easily to statistical inferencing. Generalized additive models and multivariate adaptive regression splines were included because these models relax some of the rigid assumptions associated with generalized linear models, which allows them to achieve higher predictive accuracy compared to the GLM. A number of tree-based models were included, namely because previous studies have leveraged these algorithms for hydrological applications. Beyond hydrological modeling, tree-based models are widely popular in many different areas because they generally capture the structure of the data well, have an intuitive structure, and lend themselves to interpretations. Regression trees are generally thought of as ‘low-bias, high-variance’ techniques, meaning while they capture the structure of the data (i.e., they have a low bias), they are not stable and minor perturbations of input data can lead to significantly different tree structures (i.e., they have a high variance). To reduce the variance of tree-based models and improve their stability, meta-algorithms such as boosting and bagging (i.e., bootstrap aggregation) can be leveraged to improve the predictive performance. Bagging trees, as done in the bagged CART model, consists of taking bootstrap samples of the input data and developing a tree model for each sample and then aggregating all of the trees. However, while model averaging is an effective variance reduction technique, its effectiveness is limited if the aggregated trees are correlated to one another. The random forest algorithm addresses this limitation by adding another layer of randomness to the model through randomly sampling a subset of variables for each tree, which reduces the correlation among the trees. Random forest is therefore a low-bias, low-variance technique that yields robust estimates, even in the presence of outliers and noise. The Bayesian additive regression tree method is another robust ensemble-of-trees approach, where the meta-algorithm boosting is applied to the trees. Boosting differs from bagging in that each tree is used to fit the unexplained variability of the previous tree, ultimately improving the final model’s variance. Finally, the support vector machine is a theoretically grounded and powerful machine learning algorithm that leverages hyperplanes to classify the feature space by maximizing the distance between the nearest training data points of any class to the hyper-plane (boundary). To account for non-linearity, the algorithm uses kernel functions to project the non-linear feature space to higher dimensions; using kernel functions, however, significantly reduces the interpretability of the model (particularly in a regression setting). Detailed theoretical foundations and mathematical formulations of the above-mentioned methods are included in the Supplementary Methods. It should be noted that there is a host of other flexible, non-parametric machine learning algorithms such as artificial neural networks and generic programming that can account for non-linearities in the data. However, since the goal in this paper is not only prediction, but also making statistical inferencing, such models fall outside the scope of the present analysis. More specifically, while methods such as artificial neural networks can provide robust predictions, due to the transformations of the input space in the inner layers, statistical inferencing cannot be easily implemented^19^.

Using 5-fold cross-validation, each model was iteratively trained with 80% of the data and then tested on the remaining 20%. The main performance assessment was based on the root-mean-squared error (RMSE), which was performed for both in-sample (training data) and out-of-sample (test data) and averaged over the five iterations. The results from this initial analysis can be found in Table 1. Here, a lower RMSE indicates that the model was better able to predict the reservoir level. However, it is important to note that the in-sample RMSE is the result of predicting values that have already been used to train the model, and therefore, is biased and may not be an accurate measure of the predictive accuracy of the model. For this reason, the out-of-sample RMSE (which accounts for the data not used in the training process) is the preferred measure of predictive accuracy.

**Table 1:** Results from the initial performance analysis of the models.

Model	In-Sample RMSE	Out-of-Sample RMSE
GLM	3.83	3.83
GAM	3.83	3.16
MARS	3.38	3.41
CART	3.29	3.32
Bagged CART	3.18	3.21
Random Forest	0.66	1.45
SVM	0.64	1.91
BART	1.97	2.04
Null (Mean-Only)	4.59	4.59

### Model selection

**Figure 1:**
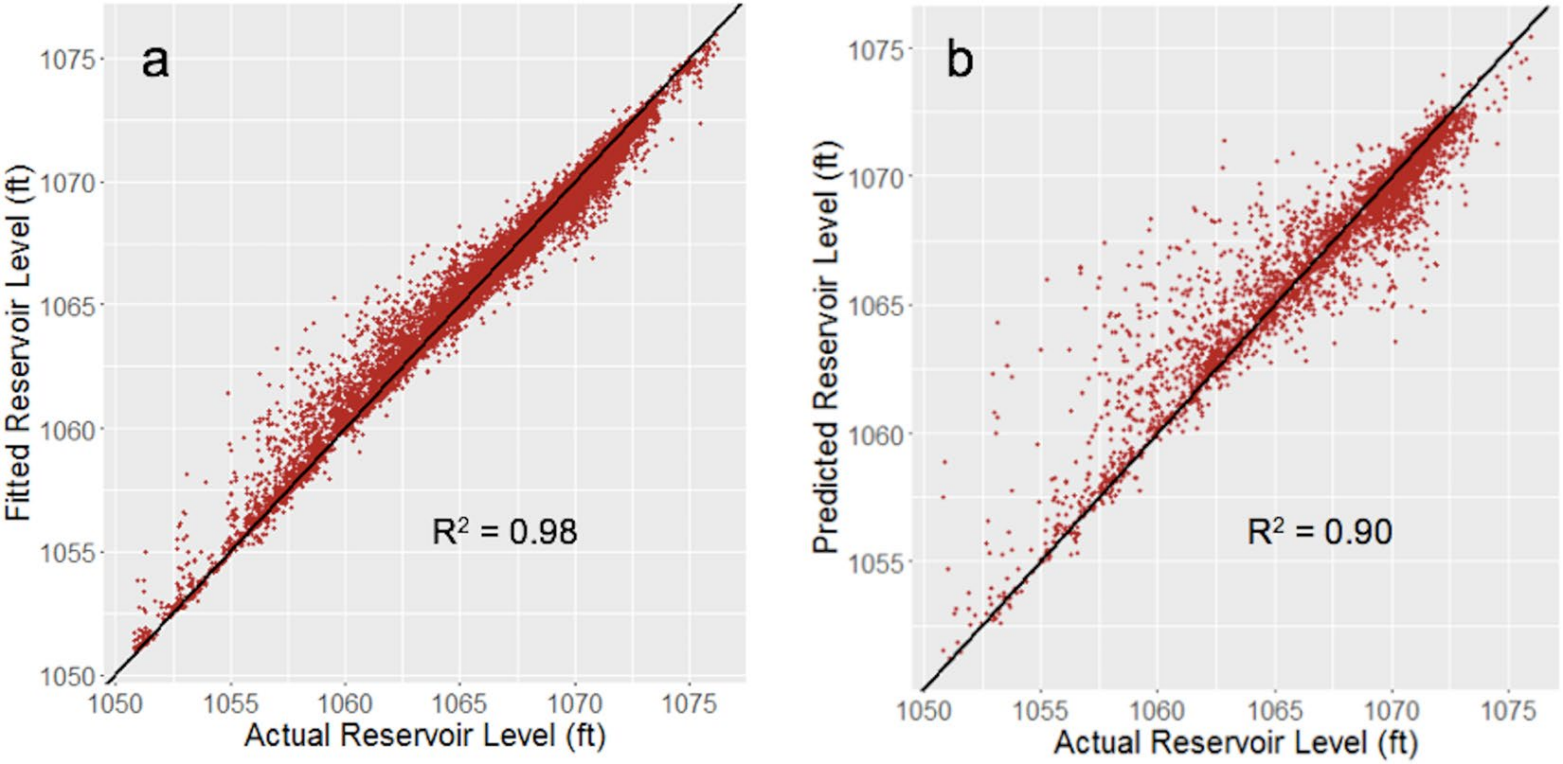
Actual reservoir levels compared to (**a**) the fitted values and (**b**) the predicted values. A 45-degree line has been plotted for reference.

The random forest model had the lowest out-of-sample error (1.45) but was closely followed by the support vector machine (RMSE of 1.91) and Bayesian additive regression tree (RMSE of 2.04) models. Therefore, before selecting the random forest model as the final model, we ran two pairwise t-tests to determine if the differences between the models (i.e., random forest vs. SVM and random forest vs. BART) were statistically significant. A Shapiro-Wilk test was performed to confirm the normality of the data prior to performing the t-tests. The results of the t-tests confirmed that the random forest model outperformed the other models in a statistically significant way. That is, both tests demonstrated that the differences between the random forest RMSE and the other RMSE values were statistically significant. Specifically, the t-test between random forest and SVM had a p-value of 1.364 × 10^−5^ and the t-test between random forest and BART has a p-value of 2.396 × 10^−4^. It is interesting to note that random forest outperformed the more theoretically grounded and complex models (i.e., SVM and BART). This is can be explained by the bias-variance tradeoff—as bias decreases, variance increases. It is the goal in statistical learning is to simultaneously minimize both bias and variance. Complex methods tend to do well at minimizing bias, but not variance. Therefore, the best model may be a less complex model that is better able to minimize variance without losing too much accuracy to the increase in bias. Random forest, as described in the previous section, was designed to minimize the bias and variance, making it a powerful predictive model, even when compared to SVM and BART. The random forest model also showed an improvement of 68% over the null model (i.e., the mean-only model), demonstrating the ability of the model to predict reservoir level beyond the historical averages. This supports our initial hypothesis that the random forest model would perform the best. In addition to having a small error, which demonstrated high predictive accuracy, the random forest model also had a high goodness-of-fit, as demonstrated in Fig. 1. This figure shows the actual reservoir levels plotted against the fit of the training data (a) and the predicted values of the test data (b), with a 45° line for reference.

### Variable importance in the random forest model

**Figure 2:**
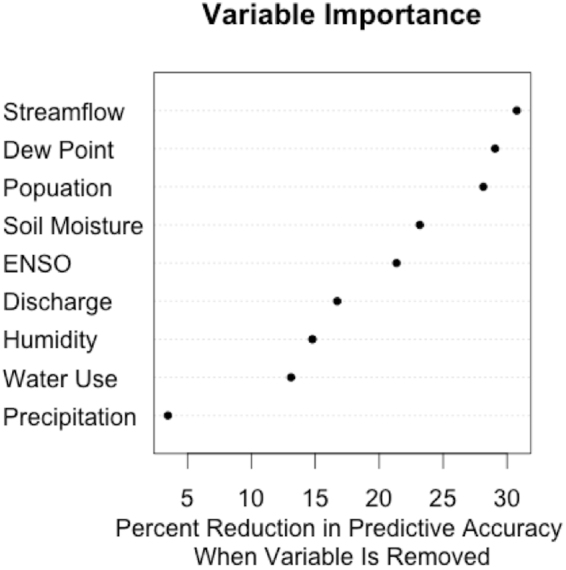
Predictors ranked by importance in the random forest model. The higher values represent higher contribution to predictive accuracy.

In addition to knowing which model performs the best, it is important to understand which predictors are contributing the most to the predictive accuracy. That is, which predictors most greatly affect the reservoir level. Variable importance is measured by ranking the predictors based on their contribution to the out-of-sample accuracy. That is to say, the larger the decrease in accuracy after the removal of a predictor, the more important that predictor is to the final model. As shown in Fig. 2, the most important variables were the streamflow (into the reservoir), dew point temperature, and population, followed by soil moisture and the El Niño/Southern Oscillation (ENSO) index. Conversely, precipitation was the least important variable when trying to predict reservoir level. Therefore, one could remove precipitation from the model and not lose significant predictive accuracy. In fact, the predictive performance of the model may increase, since the removal of an unrelated variable will reduce the complexity of the model and improve the bias-variance trade-off.

### Partial dependencies of variables in the random forest model

Partial dependencies are a useful measure for assessing the relationship between the individual predictors and the response variable in nonparametric models^20^. In this project, the partial dependencies were calculated using equation 1, as described by Friedman *et al*.^7^.

1 $$\bar{f}(x)=\frac{1}{n}\sum _{i=1}^{n}f(x,{x}_{iC})$$

where *x* is the variable of interest and *x*_*iC*_ represents the other variables.

**Figure 3:**
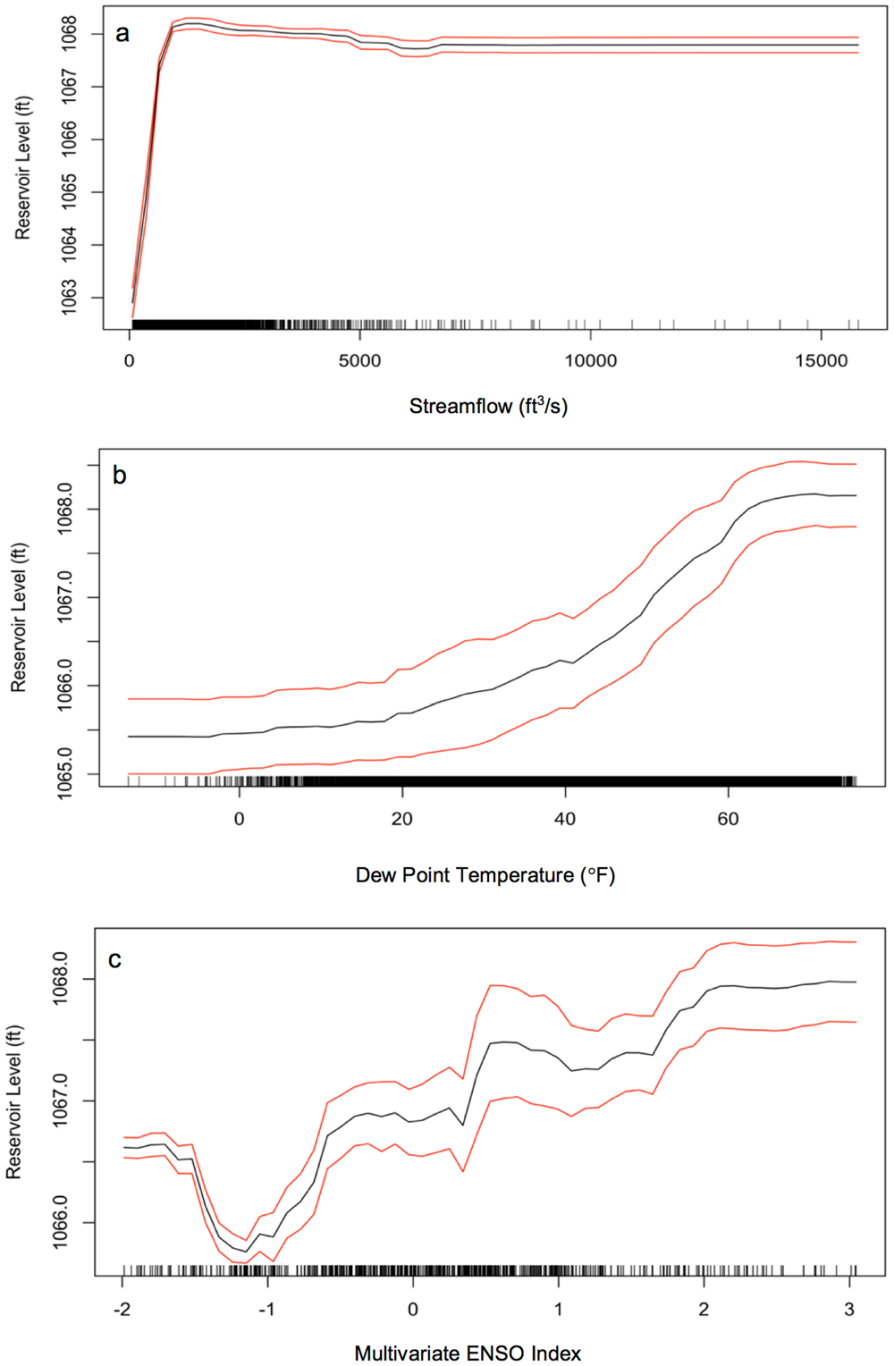
Partial dependency plots for the (**a**) streamflow into Lake Lanier, (**b**) dew point temperature, and (**c**) multivariate ENSO index, each with a 95% confidence band and data distribution notches along the x-axis.

Results from the partial dependency analysis can be used to determine the effects of individual variables on the response, without the influence of the variables. In the case of the streamflow into Lake Lanier, which was the most important variable in predicting the reservoir level, the partial dependence plot for the streamflow in Atlanta is as expected (see Fig. 3a). Low streamflow means low reservoir levels, but there is a point in which additional streamflow does not influence the water level. This threshold is near the capacity of the reservoir (around 1070 feet), so it is indicative that the managers are releasing water to keep the level at a manageable level. Another important variable was the dew point temperature. The dew point temperature is the temperature at which the air is fully saturated with water vapor. In this study, the mean daily dew point temperature was used as a predictor. As shown in Fig. 3b, as the dew point increased the reservoir level also increased. A higher dew point is indicative of more moisture in the air, leading to less evaporation and more water staying in the reservoir. In this sense, water managers can assess the state of their water resources by evaluating the streamflow and the mean dew point temperature—a low streamflow with high dew point might not be too damaging, but a low streamflow and low dew point could be cause for concern. Finally, the ENSO intensity was also a relatively important variable in the random forest model. The partial dependence plot can be seen in Fig. 3c, where there is strong trend towards the presence of an El Niño leading towards higher reservoir levels. The effects of an El Niño in the southeastern United States are increased precipitation and cooler temperatures^21^, which would ultimately lead to more water entering and staying in the reservoir.

### Comparison of results to other cities

**Figure 4:**
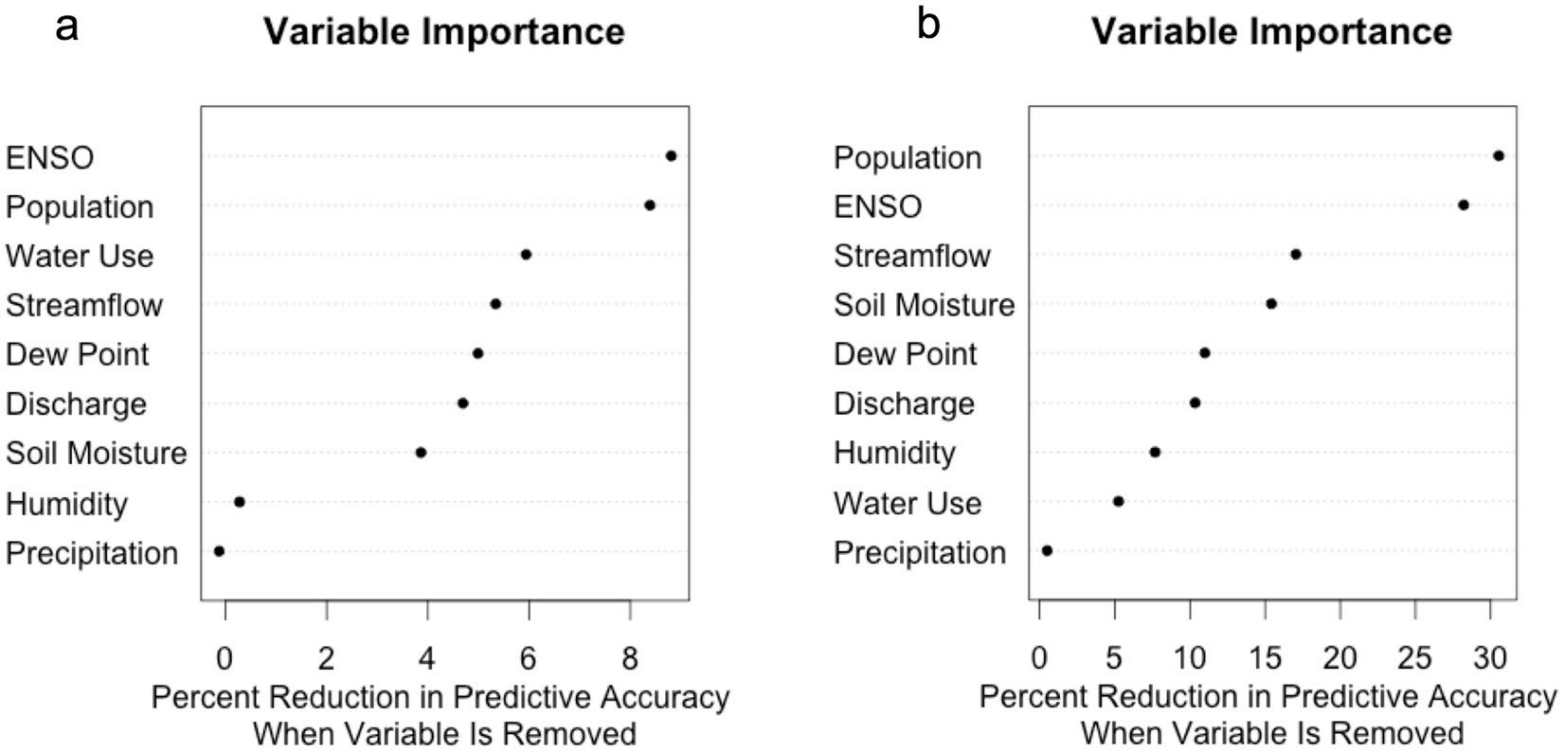
Variable importance plots for the random forest models run for (**a**) Eagle Creek (Indianapolis, IN) and (**b**) Lake Travis (Austin, TX).

As we demonstrated above, the random forest model was the best model for predicting the water level in Lake Lanier. However, this result may be specific to Lake Lanier. To test this site specificity, we ran the same random forest model in two other reservoirs: Eagle Creek (Indianapolis, IN) and Lake Travis (Austin, TX). Similar to Lake Lanier in Atlanta, both reservoirs serve as the main source of drinking water for their respective cities. Likewise, both regions have experienced drought years and wet years within the study period. We found that the predictions from the random forest model greatly outperformed the prediction made by the null (mean-only) model in both cases. Specifically, using the random forest model led to a 55% improvement for Eagle Creek and 92% for Lake Travis. This indicates that the use of the random forest model for predicting reservoir levels can be transferred to other cities. The main difference between the three cities was the important variables in the random forest model. As discussed earlier, the most important variables in the Lake Lanier analysis were streamflow, dew point temperature, and population. However, in the Eagle Creek reservoir, the ENSO index, population, and water use were the most important variables (see Fig. 4a). Finally, in the Lake Travis analysis, population, ENSO index, and streamflow were the most important variables (see Fig. 4b). This shows that although the random forest model is transferrable between different cities, there is still a need for site-specific studies to determine the important predictors.

## Discussion

This study focused on determining the most accurate statistical learning technique for predicting reservoir levels based on the current hydroclimatic conditions. We hypothesized that random forest would perform well, as tree-based methods have been successful in predicting reservoir conditions^5,6^. The results support this hypothesis and extend the practice of applying supervised learning techniques to long-term analyses, especially those focusing on drought. Though initially focused on Lake Lanier in Atlanta, our results were generalizable in two other cities with different climates and water use patterns. This indicates that implementing a random forest model in urban water management scenarios can be useful, especially if one wants to understand the important variables effecting reservoir levels. That being said, our analysis showed that, although the model was the same, the important variables differed between reservoirs. Each reservoir is going to be different, therefore it is important that future studies include a site-specific analysis to determine the important variables. In Atlanta, the most important variables are streamflow and dew point temperature. This means that a deviation from normal, specifically a decrease, in either one or both variables could be indicative of a decrease in reservoir level that has the potential to develop into a hydrological drought. This is crucial information for water managers who must make decisions about drought declaration and water use restrictions. In Indianapolis and Austin, the climates are different than Atlanta and therefore, the important variables are different. Specifically, both reservoir levels in Indianapolis and Austin have a high dependency on population and ENSO. The relative importance of population in all three cities is likely because as the cities grow, more water is consumed, even in the presence of conservation measures. The ENSO index is another variable that affects all three cities, although it is more important in Indianapolis and Austin. The ENSO is a large-scale climate process that is formed in the Pacific Ocean when there are major shifts in sea surface temperature. Although the ENSO occurs in the Pacific Ocean, it has major effects on the climate around the world, including changes in precipitation over the US that may lead to drought. We used the NOAA Multivariate ENSO Index (MEI) to describe ENSO intensity. This index runs from −2 to 3, with more negative values indicating a strong La Niña and more positive values indicating a strong El Niño^22^. The effect of ENSO on reservoir levels is an important one, as it is a predictable climatic phenomenon, and therefore, knowing the effects on water availability could greatly impact a city’s ability to prepare for a potential drought. Interestingly, in all three cities, precipitation was the least important variable. This is likely because we used daily precipitation data to predict the reservoir level of that same day. It is more likely that a weekly accumulation of precipitation will have a greater impact on the reservoir level than the precipitation that day. Overall, this study demonstrated the ability of the random forest model to accurately predict reservoir levels, given the current hydroclimatic conditions and city characteristics, for three different cities. In the future, we plan on including antecedent precipitation and a time series aspect as well as expanding the study to more cities around the world.

## Data and Methods

### Site description

The main focus for this study was Atlanta, Georgia, although Indianapolis, Indiana and Austin, Texas were also included in the analysis. The city of Atlanta obtains nearly 90% of its water from Lake Sidney Lanier^23^, a reservoir located northeast of the city on the Chattahoochee River. Atlanta itself is located in the northern part of the state of Georgia, which is in the southeastern United States. For a visual depiction of the location of the city and reservoir, see Supplementary Fig. S1. Atlanta is in a semi-humid climate zone, yet the region regularly experiences severe droughts that are accompanied by drops in reservoir levels^24^. Atlanta is a major metropolitan area in the United States, currently home to over 470 thousand people within the city limits and 5.7 million people in the metropolitan area^25^. The Atlanta population is heavily dependent on Lake Lanier for its drinking water, making it imperative that it is secured for the future, a task which is complicated by the water laws in the Chattahoochee River basin^24^. If the water managers in charge of Atlanta’s water supply have knowledge on the hydroclimatic conditions that may lead to reduced water supply, they can better prepare while maintaining adequate supply downstream. The relative dependence on a single source as well as a climate that is prone to occasional severe droughts makes Atlanta an ideal location to study the viability of machine learning techniques to predict urban reservoir water levels. Additionally, the Eagle Creek reservoir, which serves the city of Indianapolis, and Lake Travis, which serves the city of Austin, were included in the analysis in order to test the generalizability of the results obtained from Atlanta. Both reservoirs, like Lake Lanier, are major sources for the cities they serve. The main difference between the cities are the climates and water usage patterns, making them ideal for studying the transferability of the results.

### Data description

Data for this study was obtained from several government agencies, including the US Army Corps of Engineers (USACE), the US Geological Survey (USGS), and the National Centers for Environmental Information (NCEI). Specifically, we obtained the reservoir level data from USACE^26^, streamflow data from USGS^27,28^, and precipitation, humidity, and temperature data from NCEI^29,30^. Additionally, we obtained population data from the US Census Bureau^25^, water use data from the North Georgia Water Planning district^23^, soil moisture from the NOAA Climate Prediction Center^31^, and ENSO data from NOAA^22^. The streamflow data was collected from two locations: one 20 miles upstream of the reservoir (USGS site 02331600) and one 30 miles downstream of the city (USGS site 02338000). The meteorological data was collected from the Atlanta Hartsfield International Airport, which maintains a long-running and accurate weather station southwest of the city (about 45 miles southwest of the reservoir). This station was selected due to the longevity of the data record and the realitive quality of the data. Although it is not exactly positioned next to the reservoir, it is close enough that most of the meteorology will not change much between the two locations. The population and water use data are both limited to the city itself, not the metropolitan area. This was done because the North Georgia Planning District specifically separated the city of Atlanta from the remainder of the district. Finally, the soil mositure was collected from the CPC, which is a gridded product. We selected the grids surrounding the reservoir and averaged them to obtain a soil moisure in the area.

**Figure 5:**
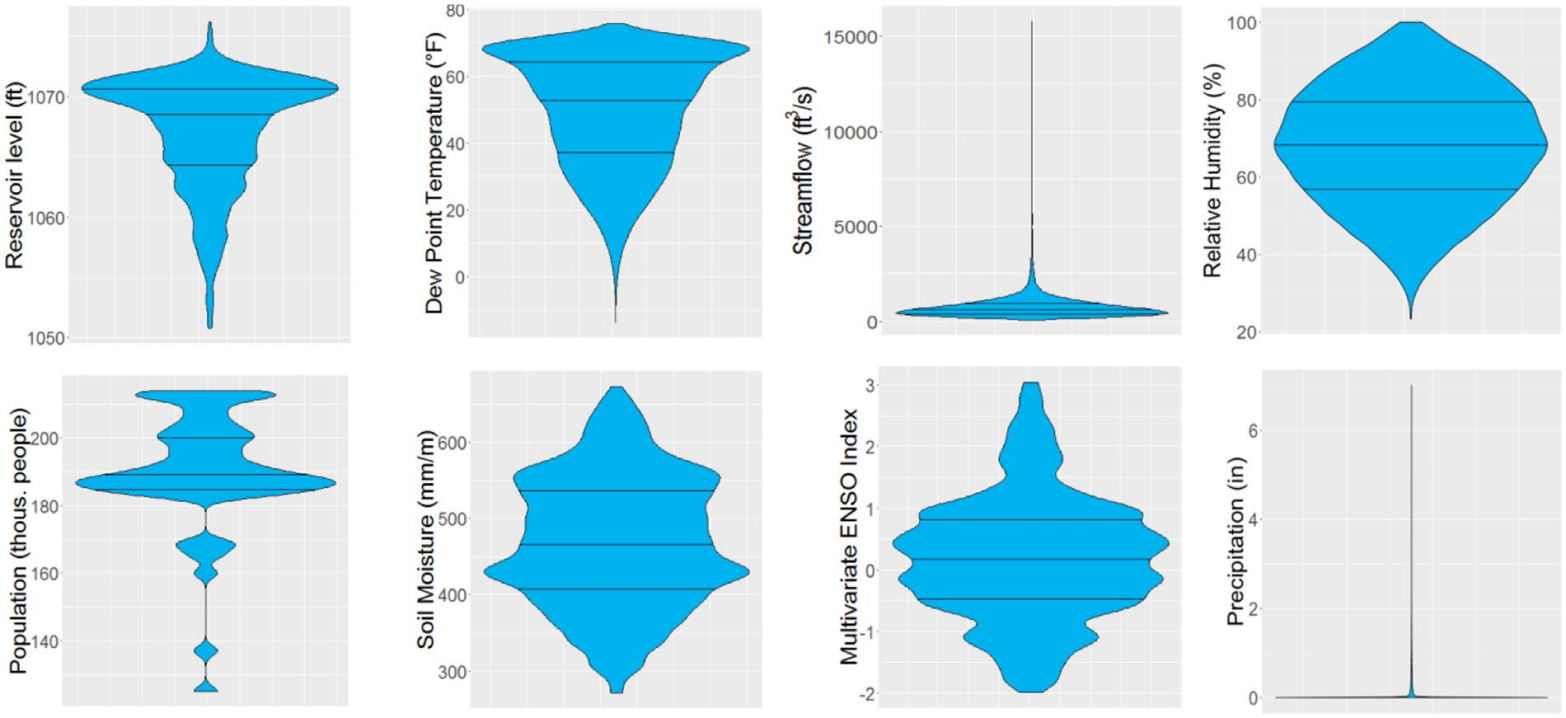
Violin plot showing the density of six variables: reservoir level, dew point, streamflow, humidity, population, soil moisture, ENSO index, and precipitation. Discharge and water use plots are not shown because they have similar patterns to the streamflow and population plots, respectively.

Our data included daily values from 1965–2016 (those that were not initially daily measurements, were scaled to that resolution). During this period, the reservoir level ranged from 1050.8 to 1076.2 feet with a mean of 1067.1 ft. Likewise, the streamflow (into the reservoir) ranged from 66 to 15800 ft^3^/s with a mean of 766 ft^3^/s, while the discharge (downstream of the city) ranged from 852 to 58600 ft^3^/s with a mean of 3900 ft^3^/s. The dew point temperature ranged from −13.6 to 75.7 °F with a mean of 49.7 °F, the relative humidity ranged from 23.3 to 100.0% with a mean of 68.0%, and the precipitation ranged from 0 to 7.0 inches with a mean of 0.13 in. Finally, the soil moisture ranged from 271.3 to 673.2 mm/m with a mean of 470.2 mm/m. The distribution of these variables and others can be seen in Fig. 5.

In this study, the response variable was the reservoir level and the predictors were: streamflow (into the reservoir), precipitation, population, water usage, discharge (downstream of the city), ENSO index, soil moisture, dew point temperature, and relative humidity. These predictors were selected based on a thorough review of the literature on the subject. Specifically, we chose to include streamflow into the reservoir, as it is the most likely determinant of reservoir level. That is, there is unlikely to be a higher reservoir level if the streamflow is lower than usual. For similar reasons, we chose to include population and water usage as predictors. Given that one of the main uses of Lake Lanier is providing drinking water for the city of Atlanta^24^, it is logical that the population using the water as well as the amount of water consumed will be important variables in predicting reservoir level. Additionally, we included the discharge downstream of the reservoir, which includes both wastewater and overflow discharges. These are related to reservoir level and it was thought that they may provide additional information about reservoir level. Moreover, the streamflow, withdrawals, and discharge were all used in previous studies by de Araújo and Bronstert^4^ and Yang *et al*.^6^ We also included a few meteorological variables, including precipitation, dew point temperature, and humidity. These variables were selected as atmospheric measures of the hydrological cycle. In other words, we wanted to include several atmospheric variables that either inherently a part of the water cycle (i.e., precipitation) or closely related (i.e., dew point temperature and humidity, which both have influence over evaporation). Atmospheric variables are easily measured and made available to the public, and, as demonstrated by Ficchi *et al*.^5^, are necessary for predicting reservoir levels. Therefore, we decided to include them in our analysis. The decision to include soil moisture as a predictor followed similar reasoning, though it is not an atmospheric variable, it does have a role in the hydrological cycle. Specifically, soil moisture is used a proxy for storage, similar to reservoir level. Therefore, it is reasonable to say that water stored in the soil is not water stored in the reservoir, which affects the water level. Finally, we included the El Niño/Southern Oscillation index because of its known effects in the southeast region of the United States^21^. The ENSO has major climate impacts across the United States, so it is likely that there will be some changes in reservoir level depending on the strength of the El Niño (or La Niña). Overall, the predictors were selected using knowledge gained from the literature review as well as that previously known to the authors. Further details can be found in Supplementary Table S1, along with a correlation matrix of the variables (Supplementary Fig. S1).

### Statistical models and analysis

Supervised learning is branch of statistical learning theory in which the response variable guides the learning process. Mathematically, supervised learning technique can be described as: y = f(X) + ε; where y represents the process of interest (the reservoir level in this study); X represents the series of input variables used to estimate the response (see Supplementary Table S1 for variable list); and the noise {ε ~ N(0, σ^2^)} represents the irreducible error^19^. The goal of supervised learning is to leverage data and estimate a statistical response surface $\hat{{\rm{f}}}({\rm{X}})$ such that the loss function ${\rm{L}}=\int {\rm{\Delta }}[\hat{{\rm{f}}}({\rm{X}}),{\rm{f}}({\rm{X}})]{\rm{dX}}$ is minimized over the entire domain of the independent variable X. Here, Δ represents a measure of distance (e.g., Euclidean distance) between the estimated and actual response functions^19^.

In this study, we employed several statistical models to predict the reservoir level based on the predictors. Specifically, we used the: (1) generalized linear model (GLM)^12^, (2) generalized additive model (GAM), (3) multivariate adaptive regression splines (MARS)^11^, (4) classification and regression trees (CART)^14^, (5) bagged classification and regression trees^15^, (6) random forest^16^, (7) support vector machine (SVM)^17^, and (8) Bayesian additive regression trees (BART)^18^ methods. As discussed earlier, these methods were chosen to ensure a variety of algorithms were tested. Descriptions and mathematical representations of these algorithms can be found in Supplementary Methods. Additionally, the final model structure for each algorithm can be found in Supplementary Table S2.

Model performance was assessed based on randomized 5-fold cross validation, such that each fifth of the data was used as a test set for the remaining data. The final error was calculated by averaging the root-mean-squared error (RMSE) of each of the folds. RMSE was chosen as the main measure of error because it penalizes larger deviations more heavily, making it a suitable choice for applications in which large prediction errors are highly undesirable. RMSE represents the out-of-sample (test data) error of the model and is calculated using equation (2).

2 $$RMSE=\,\sqrt{\frac{\sum {({x}_{P}-x)}^{2}\,}{n}}$$

where ${x}_{P}$ represents the predicted values, *x* represents the actual values, and *n* is the number of observations.

The final model was selected based on the best (lowest) RMSE, and then confirmed through a series of pairwise t-tests. In other words, we compared the results from the model with the lowest RMSE to the two models with the next lowest RMSE values. The t-tests were performed after the Shapiro-Wilk test failed to reject the null hypothesis that the data were normally distributed. The purpose of the pairwise t-tests was to determine if there was a statistically significant difference between the results of each model.

## Electronic supplementary material

Supplementary Information
