# Supplementary material for: Predicting Urban Reservoir Levels Using Statistical Learning Techniques
Source: Sci Rep. 2018 Mar 26;8:5164. doi: 10.1038/s41598-018-23509-w (PMC5980089; doi:10.1038/s41598-018-23509-w)
Supplement: Supplementary file 1 — Supplementary Information [file 41598_2018_23509_MOESM1_ESM.pdf]

Predicting Urban Reservoir Levels Using Statistical Learning Techniques

Renee Obringer[1,*], Roshanak Nateghi[2]

[1]Department of Earth, Atmospheric, and Planetary Sciences, Purdue University, West Lafayette, IN 47907, USA.
[2]School of Industrial Engineering, and Division of Environmental and Ecological Engineering, Purdue University, West Lafayette, IN 47907, USA.

[*]Corresponding Author: Renee Obringer (robringe@purdue.edu)

Contents of this file include:

Methods
Tables S1 and S2
Figures S1 and S2

Methods

Generalized Linear Model (GLM)

The generalized linear model (GLM) is an extension of linear regression that relaxes the normality assumption. In this model, the response is generated from an exponential distribution and then related to the predictors through a link function[1]. The GLM is defined by:

I. A dependent variable Y that has a known distribution (i.e., normal, binomial, Poisson, or gamma), as shown below:

$$Y_i \sim f_{Y_i}(y_i)$$

$$f_{Y_i}(y_i) = exp\left[\frac{y_i\theta_i - b(\theta_i)}{a(\phi)} + c(y_i, \phi)\right]$$

where θ and ϕ are the location and scale parameters, respectively.

II. A set of independent variables x_i.

III. A linking function $g(.)$ that relates the response variable to the predictors.

Generalized Additive Model (GAM)

The generalized additive model (GAM) is a further extension of linear regression, which in addition to relaxing the normality assumption as in the GLM also relaxes the linearity assumption, meaning that there could be local nonlinearities[2]. In the GAM, the response variable y has a distribution with mean $\mu = E[Y|x_1, x_2, \dots, x_n]$ that is linked to the predictors through the link function:

$$g(\mu_i) = \alpha + \sum_{j=1}^{n} f_j(x_j)$$

where f_j is a smoothing function (i.e., a regression spline).

Multivariate Adaptive Regression Splines (MARS)

The multivariate adaptive regression splines (MARS) method is a semi-parametric procedure that combines recursive partitioning regression and spline fitting[3]. The model takes on the following mathematical form:

$$f(X) = \beta_0 + \sum_{j=1}^{n} \beta_j h_j(X)$$

where $h_j(X)$ is the linear spline, β_0 is the intercept, and β_j is the vector of coefficients, which are estimated by minimizing the sum of squares error. The MARS method also uses generalized

cross validation (GCV) to avoid overfitting the model. This method penalizes complexity, which makes MARS especially applicable for high-dimensional datasets.

$$GCV = \frac{RSS}{N \times \left(1 - {}^{C}/_{N}\right)^{2}}$$

where RSS is the residual sum of squares, N is the number of observations, and C is the effective number of parameters.

Classification and Regression Trees

The classification and regression tree (CART) method operates by iteratively partitioning the dataset into boxes in such a way that the residual sum of squares is minimized[4]. The partitioning is performed using the recursive binary splitting technique, an example of which is shown below:

$$R_1(j,s) = \{X|X_j < s\} \text{ and } R_2(j,s) = \{X|X_j \geq s\}$$

where R_1 and R_2 are the partitioned boxes, X is the dataset, and s is the partitioning threshold.

Bagged Classification and Regression Trees

Bagging is a meta-algorithm that uses bootstrap aggregation to reduce the variance of the prediction[5]. The bagged CART method uses bootstrapping to iteratively run the CART method over a subset of the data, the final tree being an aggregation of all the iterations. The mathematical representation of bagging is:

$$\varphi_B(x) = av_B\varphi\left(x, \mathcal{L}^{(B)}\right)$$

where $\mathcal{L}^{(B)}$ is the subset of the data used in the bootstrapping procedure and $\varphi\left(x, \mathcal{L}^{(B)}\right)$ is the predictor formed from the bootstrapped sample. B represents the number of bootstrapped iterations.

Random Forest

Random forest is a tree-based ensemble method that builds B bootstrapped, de-correlated regression trees and then aggregates those trees to a single model[6]. The additional layers of randomness introduced in the random forest algorithm that leads to reduced correlation among the trees leads to further variance reduction and as a result improved performance over bagged-trees. The final model can be represented by the average of all the trees:

$$\hat{f}^B(x) = \frac{1}{B}\sum_{b=1}^{B} T_b(x)$$

where T_b is the regression tree and B is the number of bootstrapping iterations.

Support Vector Machines

Support vector machine (SVM) is an optimization technique, which allows for finding the global solution and therefore often leads to more accurate predictions[7]. The goal of the support vector machine algorithm is to find a hyperplane that maximizes the margin between the two classes of data. The hyperplane can be found by:

$$\widehat{f_x} = \sum_{i=1}^{n} \hat{a}_i K(x, x_i)$$

where $\hat{a}_i = (HH^T + \lambda I)^{-1} y$, a transformation of the basis matrix H and $K(x, x_i)$ is the kernel function (i.e., linear, radial, or polynomial).

Bayesian Additive Regression Trees

The Bayesian additive regression tree (BART) technique is an ensemble-based method that uses boosting to improve predictive accuracy[8]. Boosting, as opposed to bagging, fits a series of trees in which each tree is used to fit the variability not explained by the previous trees[9]. The BART method works by constraining each individual tree by implementing a regularization prior, creating a series of weak learners. The result is a sum of trees where each tree explains a different part of the whole:

$$Y = \sum_{j=1}^{n} g(x;\ T_j, M_j) + \varepsilon$$

where T_j is a single regression tree, M_j is a set of parameter values, and ε is the error with distribution $N(0, \sigma^2)$.

Table S1. Data collected for this study.

Variable Type	Variable Name	Minimum	Maximum	Mean	Units	Source
Response	Reservoir Level	1050.8	1076.2	1067.1	ft	USACE[10]
Predictor	Precipitation	0.0	7.0	0.13	in	NCEI[11]
	Streamflow	66	15800	766.3	ft^3/s	USGS[12]
	Discharge	852	58600	3900	ft^3/s	USGS[13]
	Water Use	393760	497337	432022	gpcd	North Georgia Water[14]
	Population	125	214	190	10^3 persons	US Census Bureau[15]
	ENSO	-2	3	0.20	--	NOAA[16]
	Soil Moisture	271.3	673.2	470.2	mm/m	CPC[17]
	Dew Point	-13.6	75.7	49.7	°F	NCEI[18]
	Humidity	23.3	100.0	68.0	%	NCEI[18]

Table S2. Tuning parameters used in the statistical models.

Model	Tuning Parameter*	Tuning Parameter Value	Tuning Parameter Description
GLM	family	Gaussian	Link function
GAM	Stepwise update	--	Runs through model in stepwise fashion
MARS	nk	8	Maximum number of model terms
	nprune	7	Maximum number of terms in pruned model
	degree	1	Degree of interaction
	penalty	1	GCV penalty per knot
CART	--	--	--
Bagged CART	nbagg	25	Number of bootstrap replications
Random Forest	ntree	30	Number of trees to grow
	mtry	4	Number of variables to sample at each split
SVM	kernel	radial	Kernel used in training and predicting
	cost	10	Cost of constraint violation
	gamma	1	Required parameter for radial kernels
BART	num_trees	10	Number of trees to grow**
	num_burn_in	20	Number of samples to be discarded as “burn-in”
	q	0.99	Quantile of the prior
	k	1	Determines the prior probability
Null	--	--	--

*Note: The names of the tuning parameters are specific to the packages used in R and may be different in other programming languages or libraries.
**Note: BART is computationally expensive, so this value had to be constrained due to memory limitations.

Figure S1. Map showing the location of the city of Atlanta and Lake Sidney Lanier. The yellow star indicates the city and the blue star indicates the reservoir. Imagery: Landsat/Copernicus. Map data: Google.

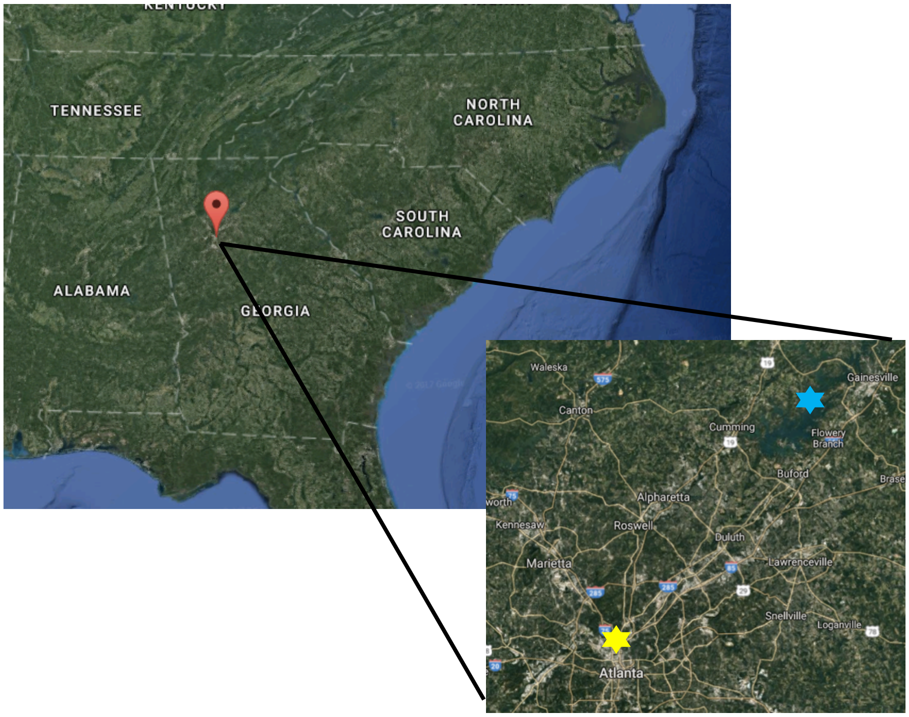

Figure S2. Correlation matrix of variables used in study.

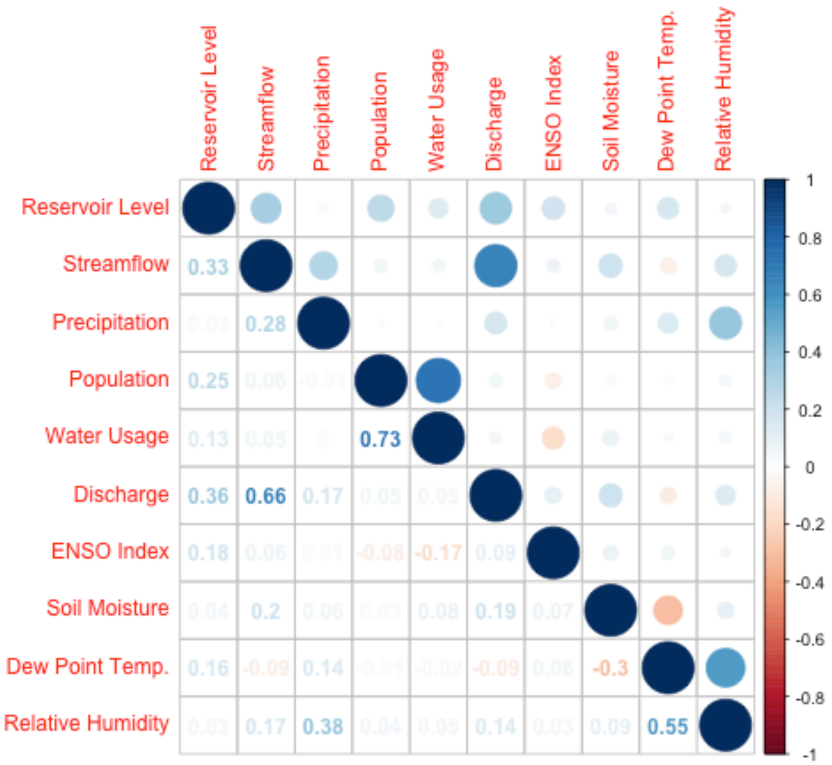

References

1. Nelder, J. A. & Wedderburn, R. W. M. Generalized Linear Models. *J. R. Stat. Soc. A.* **135,** 370–384 (1972).
2. Hastie, T. & Tibshirani, R. Generalized Additive Models. *Stat. Sci.* **1,** 297–318 (1986).
3. Friedman, J. H. Multivariate Adaptive Regression Splines. *Ann. Stat.* **19,** 1–67 (1991).
4. Breiman, L., Friedman, J. H., Olsen, R. A. & Stone, C. J. *Classification and Regression Trees Ch. 8,* 216–264 (Wadsworth International Group, 1984).
5. Breiman, L. Bagging Predictors. *Mach. Learn.* **24,** 123–140 (1996).
6. Breiman, L. Random forests. *Mach. Learn.* **45,** 5–32 (2001).
7. Cortes, C. & Vapnik, V. Support Vector Networks. *Mach. Learn.* **20,** 273–297 (1995).
8. Chipman, H. A., George, E. I. & McCulloch, R. E. BART: Bayesian additive regression trees. *Ann. Appl. Stat.* **6,** 266–298 (2012).
9. Friedman, J. H. Greedy Function Approximation: A Gradient Boosting Machine. *Ann. Stat.* **29,** 1189–1232 (2001).
10. United States Army Corps of Engineers. Lake Lanier Level Data. (2017).
11. NOAA National Centers for Environmental Information. Hourly Precipitation Data. (2016).
12. United States Geological Survey. USGS 02331600 Chattahoochee River Near Cornelia, GA. (2017).
13. United States Geological Survey. USGS 02338000 Chattahoochee River Near Whitesburg, GA. (2017).
14. Atlanta Regional Commission. *Metropolitan North Georgia Water Planning District Water Metrics Report*. (2011).
15. United States Census Bureau. QuickFacts: Atlanta city, Georgia. (2016).
16. Wolter, K. & Timlin, M. S. Measuring the strength of ENSO events: How does 1997/98 rank? *Weather* **53,** 315–324 (1998).
17. Dool, H. van den, Huang, J. & Fan, Y. Performance and Analysis of the constructed analogue method applied to US soil moisture applied over 1981-2001. *J. Geophys. Res.* **108,** 1–16 (2003).
18. NOAA National Centers for Environmental Information. Local Climatological Data (LCD). (2010).
